# OPN and αvβ3 Expression are Predictors of Disease Severity and Worse Prognosis in Hepatocellular Carcinoma

**DOI:** 10.1371/journal.pone.0087930

**Published:** 2014-02-03

**Authors:** Yi Jin, Jian-ning Chen, Zhi-ying Feng, Zhi-gang Zhang, Wen-zhe Fan, Yu Wang, Jia-ping Li

**Affiliations:** 1 Department of Pathology, the Third Affiliated Hospital, Sun Yat-Sen University, Guangzhou, China; 2 Department of Interventional Oncology, the First Affiliated Hospital, Sun Yat-Sen University, Guangzhou, China; INRS, Canada

## Abstract

Expressions of OPN and αvβ3 are associated with a poor prognosis in many malignancies. However, their relationship in hepatocellular carcinoma remains unclear. We systematically collected hepatocellular carcinoma tissue samples from 305 patients over 3 years, and analyzed the status of OPN and αvβ3 in hepatocellular carcinoma and correlate expression with patient disease status and survival outcome. Our study results indicated that OPN and αvβ3 are expressed at significantly higher rates in hepatocellular carcinoma compared with adjacent non-tumorous tissue (69.5% vs 18.4%, p<0.01 and 77.4% vs 21.6%, p<0.01, respectively). Both OPN and αvβ3 expression levels are associated with poor prognostic factors, including tumor size, capsular invasion, tumor thrombus of the portal vein, metastasis of the lymph node and clinical staging. Patients expressing OPN and αvβ3 had significantly shorter survival compared with patients negative for protein expression (p<0.01). Multivariate analysis also showed that both OPN and αvβ3 expression are independent prognostic factors for poorer survival in hepatocellular carcinoma. By this study, we conclude that OPN and αvβ3 are negative prognostic predictors in patients with hepatocellular carcinoma. The expressions of both OPN and αvβ3 are associated with worse survival outcome.

## Introduction

Hepatocellular carcinoma (HCC) is the fifth most common cancer and the third most common cause of cancer-related deaths worldwide. At the time of diagnosis, the majority of patients are already in the middle-advanced stage, and the 5-year survival rate is very low [1], [2], [3]. Thus, early diagnosis and treatment are particularly important to improve the survival of patients with HCC. Hepatocarcinogenesis is considered to be a multi-gene and multi-stage disease process and likely involves various pathogenic factors.

Osteopontin (OPN) is an arginine-glycine-aspartate (RGD)-containing adhesive glycoprotein that was first identified as a major sialoprotein in bone and subsequently found to be expressed in kidney, brain, dentin, cementum, hypertrophic cartilage, and so on [4], [5]. OPN is expressed in a variety of cell types, including activated macrophages, lymphocytes, vascular smooth muscle cells and many cells of epithelial linings [4], [5]. Moreover, OPN also may appear in biological fluids, including blood, urine and so on [6], [7]. OPN is a multifunctional secreted phosphorylated glycoprotein that promotes cellular chemotaxis, adhesion and migration and is associated with the occurrence, metastasis and prognosis of a variety of cancer types [8], [9], [10].

Recent studies have indicated that OPN is involved in HCC progression and metastasis through its interaction with the αvβ3 (alphavbeta3) integrin receptor [11]. The αvβ3 integrin is a family of transmembrane adhesion receptors, composed of noncovalently linked α and β subunits. Recent findings indicate that the αvβ3 integrins are expressed in multiple cell types including invasive tumor cells, osteoclasts, activated endothelial and smooth muscle cells, platelets, megakaryocytes and macrophages [12]. The αvβ3 integrin plays an important role in a number of physiological and pathological processes such as bone resorption, wound healing, angiogenesis, tumor invasion and metastasis. To the same extent, it has been shown that overexpression of αvβ3 is related to tumor progression [13].When αvβ3 is activated by stimulating factors, it can bind to enhancer or promoter sequences to regulate the activation and transcription of target genes stimulating inflammation and controlling cell proliferation.The heparin-binding αvβ3 integrin mediates cell-cell and cell-matrix adhesion, regulates intracellular signaling pathways and induces the activation of protein-dissolving enzymes, thereby promoting the invasion and migration of tumor cells [14]. Previous studies found that αvβ3 is detected in a variety of tumor types and is closely associated with both tumor development and the degree of tumor malignancy [14]. Reportedly, OPN acts through αvβ3 integrin, which in turn activates the FAK, PI3K, Akt, ERK, NF-κB and Pim-1 pathways, thus contributing to the migration of tumor cells [15]. Concurrently, these results suggest that OPN mediates migration in human cancer cells via the αvβ3 integrin signaling pathways. The expression of OPN and αvβ3 in cancer are closely related. We reported that the OPN and αvβ3 proteins were frequently overexpressed in Non-small cell lung cancer (NSCLC) and were associated with some clinicopathologic variables that are of known prognostic importance in NSCLC [16]. We also showed that forced expression of MAT1A affects downstream pathways of OPN signaling, namely ERK and AKT activation in Huh7 cells [17]. To date, however, the expression dynamics of OPN, αvβ3 in HCC and their potential biological roles in the tumorigenesis of HCC have not been fully elucidated, the co-expression of these proteins and association with HCC pathogenesis have not been reported. Therefore, we chose to further investigate OPN and αvβ3 integrin, two components of these signaling pathways in the present study.

Chuan-Hai Zhang has also shown that OPN be a predictor of HCC prognosis and a meta-analysis was recently published [18]. However, we employed immunohistochemistry to further investigate OPN correlation with αvβ3 integrin expression, in the largest group of patients with HCC in the literature, and deeply explored their relationship with clinical pathological features in HCC and adjacent non-tumorous tissue. Our study indicates that analysis of OPN and αvβ3 expression may provide novel diagnostic insights in patients with HCC and provides the basis for identifying new therapeutic targets.

## Materials and Methods

### Ethics Statement

This study was approved by the Clinical Research Ethics Committee of the First Affiliated Hospital, Sun Yat-sen University. Written informed consents were taken from all the participants and ethical guidelines under Declaration of Helsinki were followed.

### Clinical Data

Specimens were obtained from archived, paraffin-embedded tissue sections from 305 patients with HCC at the First Affiliated Hospital, Sun Yat-sen University, (Guangzhou, China) between January 1^st^ 2004 and December 31^st^ 2006. Histopathological diagnosis of all specimens was confirmed by a trained pathologist. The patient cohort included 267 men and 38 women with a median age of 55 years (range 19–78 years). Patients did not have a previous history of chemotherapy or radiotherapy. A total of 305 normal liver tissue specimens were obtained from the periphery of the cancer site and utilized as controls. All patients were followed until December 2009, and the survival time was calculated from the date of surgery until death in cases of patient death. The survival times for patients who remained alive at the end of the last follow-up were regulated as censored values, and the follow-up time was 3 years.

### Protein Expression of OPN and αvβ3 in HCC

Immunohistochemistry for OPN and αvβ3 expression was performed using the streptavidin-peroxidase (S-P) kit (Maixin Biotechnology Co, Fuzhou, China). All sections were routinely deparaffinized and rehydrated, and sections were then rinsed in phosphate-buffered saline (PBS, pH = 7.4) and subsequently treated for antigen retrieval. Sections were treated in an EDTA buffer (pH = 8.0) in an autoclave sterilizer. After cooling to room temperature for 20 min, the sections were rinsed in PBS and immersed in 3% H_2_O_2_ for 15 min to block the endogenous enzymes. After rinsing in PBS, the sections were incubated with normal goat serum at 37°C for 15 min to block non-specific antibodies. After incubation with the ready-to-use rabbit anti-human polyclonal OPN antibody (Maixin Biotechnology Co) and the ready-to-use rabbit anti-human polyclonal αvβ3 antibody (Maixin Biotechnology Co), sections were rinsed in PBS and incubated with biotinylated secondary antibodies. After washing in PBS, sections were incubated with streptavidin-HRP. Sections were then rinsed with PBS and expression was visualized by incubation with 3,3′-diaminobenzidine and counterstaining with hematoxylin. Sections were dehydrated, rendered transparent, covered with coverslips and sealed with neutral gum. PBS was substituted for the primary antibody and used as a negative control.

### Assessment of OPN and αvβ3 Expression in HCC Patient Specimens

Tumor and normal tissue specimens were assessed by two independent pathologists. Histologically, HCC is characterized by trabecular growth of three or more cells in thickness, and also has a gland-like pattern and pseudoglandular pattern, admixed with the trabecular pattern. Tumor cells show marked variation in cellular and nuclear size, shape and staining. Tumor cells have abundant eosinophilic cytoplasm and round nuclei with distinct nucleoli. Adjacent non-tumorous hepatic tissue is typically composed of benign hepatocytes arranged in regular plates, usually one or at most two cells thick. Hepatocytes have cytoplasm that may be normal, clear and without nuclear atypia and mitoses, which are different from those of hepatocellular carcinoma. Simultaneously, we used marker of proliferation, Ki-67, for HCC tumor cells to show the OPN and αvβ3 expression quantified are on tumor cells but no other cell types. The Ki-67 immunohistochemical staining is predominantly in the nucleus. Ki-67 index is higher in HCC but lower in normal hepatic tissues.

OPN expression was localized predominantly in the cytoplasm and αvβ3 expression was observed mainly in the membrane or cytoplasm. Sections were considered positive if expression was detected in more than 10% of the cells in the tumor or normal tissue as previously described [19], [20].

### Statistical Analysis

Data were analyzed using SPSS version 13.0. (Chicago, IL, USA). The relationship between the expression of OPN and αvβ3 and the clinico-pathological parameters was evaluated by χ2 analyses. The correlation between two variables was evaluated by the Spearman rank correlation test. Cox regression model was used for multivariate analysis. A value of *P*<0.05 was considered statistically significant.

## Results

### Distinguishing HCC Tumor Cells from Normal Hepatocytes and other Cell Types

**Figure 1:**
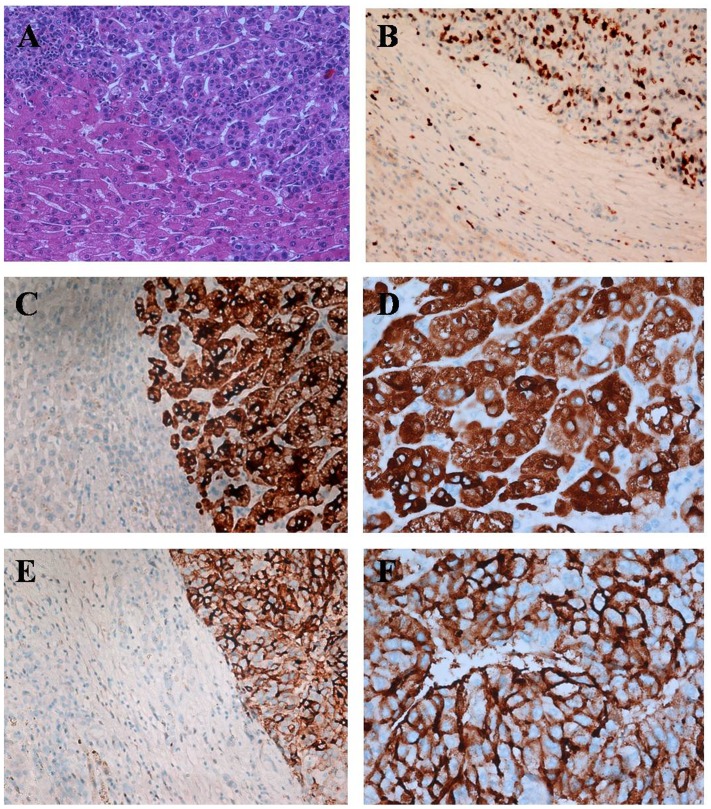
Positive expression of OPN and αvβ3 protein in hepatocellular carcinoma (HCC). (A) HE staining. The nuclei of tumor cells are large, and have atypia, which are different from those of adjacent non-tumorous hepatocytes (HE 20×10). (B) Ki-67 immunohistochemical staining. Ki-67 expression is observed in the nucleus and is visualized as brown-yellow staining. Ki-67 index is higher in tumor but lower in adjacent non-tumorous hepatic tissues (SP 20×10). (C&D) OPN expression is predominantly observed in the cytoplasm and is visualized as brown-yellow staining. The expression of OPN is negative in adjacent non-tumorous tissue (C: SP 20×10, D: SP 40×10). (E&F) αvβ3 immunohistochemical staining. Expression of αvβ3 is observed predominantly in the membrane or cytoplasm and is visualized as brown-yellow staining. The expression of αvβ3 is negative in the adjacent non-tumorous tissue (E: SP 20×10, F: SP 40×10).

In HE slices, the HCC tumor cells have large and atypical nuclei and mitosis, which are different from those of normal hepatocytes, immune cells and other cell types (Figure 1A). Next, the Ki-67 index is higher in HCC but lower in normal hepatic tissues. Although many tumor infiltrating immune cells also highly express Ki67, they can be easily distinguished because HCC tumor cells are bigger than tumor infiltrating immune cells (Figure 1B).

### Relationship between OPN Expression and Clinico-pathological Parameters in HCC

Immunohistochemical analysis of OPN in HCC specimens revealed expression predominantly in the cytoplasm (Figure 1C, 1D). In our case series, the expression of OPN was variably positive in HCC tumor cells and normal hepatocytes, scattered positive in lymphocytes, weak and focal positive or negative in vascular endothelial cells, and negative on other cell types (Figure S1). OPN expression was positive in 69.5% (212/305) of HCC specimens, compared with 18.4% (56/305) in normal hepatic tissue. There was a significant difference in OPN expression between HCC and normal hepatic tissue (*P*<0.01) (Table 1). OPN expression was related to tumor size, capsular invasion, tumor thrombus of the portal vein, metastasis of the lymph node and clinical staging in HCC (P<0.01), while it was independent of other clinico-pathological parameters (*P*>0.05) (Table 2).

**Table 1:** The expression of OPN and αvβ3 between HCC and adjacent non-tumorous tissue.

Groups	n	OPN		αvβ3	
		negative	positive (%)	negative	positive (%)
HCC	305	93	212 (69.5)	69	236 (77.4)
Normal	305	249	56 (18.4)	239	66 (21.6)

There was a significant difference in the expression of OPN and αvβ3 between HCC and adjacent non-tumorous tissue (P<0.01).

**Table 2:** Relationship between the expression of OPN and αvβ3 and clinico-pathological parameters in HCC.

Parameter	n	OPN			αvβ3		
		Negative (%)	Positive (%)	*P*	Negative (%)	Positive (%)	*P*
Sex							
Male	267	81(30.3)	186(69.7)	0.876	61(22.8)	206(77.2)	0.805
Female	38	12(31.6)	26(68.4)		8(21.1)	30(78.9)	
Age(years)							
<50	143	42(28.7)	102(71.3)	0.578	31(21.7)	112(78.3)	0.711
≥50	162	52(32.1)	110(67.9)		38(23.5)	124(76.5)	
Tumor size(cm)							
<5	126	68(54.0)	58(46.0)	0.000	30(23.8)	96(76.2)	0.678
≥5	179	25(14.0)	154(86.0)		39(21.8)	140(78.2)	
Tumor numbers							
Single	114	36(31.6)	78(68.4)	0.750	22(19.3)	92(80.7)	0.284
multitude	191	57(29.8)	134(70.2)		47(24.6)	144(75.4)	
AFP(µg/L)							
<400	145	47(32.4)	98(67.6)	0.488	34(23.4)	111(76.6)	0.743
≥400	160	46(28.7)	114(71.3)		35(21.9)	125(78.1)	
HBsAg							
positive	269	80(29.7)	189(70.3)	0.436	61(22.7)	208(77.3)	0.951
negative	36	13(36.1)	23(63.9)		8(22.2)	28(77.8)	
Capsular invasion							
have	131	15(6.1)	116(88.6)	0.000	26(19.8)	105(80.2)	0.315
no	174	78(44.8)	96(55.2)		43(24.7)	131(75.3)	
Tumor thrombus							
have	69	5(7.2)	64(92.8)	0.000	4(5.8)	65(94.2)	0.000
no	236	88(37.3)	148(62.7)		65(27.5)	171(72.5)	
Lymph metastasis							
have	91	9(9.9)	82(90.1)	0.000	6(6.6)	85(93.4)	0.000
no	214	84(39.2)	130(60.8)		63(29.4)	151(70.6)	
Pathological grade							
high	68	23(33.9)	45(66.2)	0.476	17(25.0)	51(75.0)	0.437
moderately	175	51(29.1)	124(70.9)		40(22.9)	135(77.1)	
low	62	19(30.6)	43(69.4)		12(19.4)	50(80.6)	
Hepatic function							
Child A	175	55(31.4)	120(68.6)	0.680	41(23.4)	134(76.6)	0.696
Child B	130	38(29.2)	92(70.8)		28(21.5)	102(78.5)	
Clinical stage							
I+II	168	77(45.8)	91(54.2)	0.000	54(32.1)	114(67.9)	0.000
III+IV	137	16(11.7)	121(88.3)		15(10.9)	122(89.1)	

### Relationship between αvβ3 Expression and Clinico-pathological Parameters in HCC

αvβ3 was predominantly expressed in the membrane and/or cytoplasm (Figures 1E, 1F). Similar to that of OPN, the expression of αvβ3 was also variably positive in HCC tumor cells and normal hepatocytes, scattered positive in lymphocytes, weak and focal positive or negative in vascular endothelial cells, and was negative on other cell types (Figure S1). αvβ3 expression was positive in 77.4% (236/305) of HCC cases compared with 21.6% (66/305) in normal hepatic tissue. There was a significant difference in the expression of αvβ3 between HCC and normal hepatic tissue (*P*<0.01) (Table 1). The expression of αvβ3 was related to tumor thrombus of the portal vein, metastasis of the lymph node and clinical staging in HCC (*P*<0.01), while it was independent of other clinico-pathological parameters (*P*>0.05) (Table 2).

### Association of OPN and αvβ3 Expression in HCC and Clinical Prognosis

**Figure 2:**
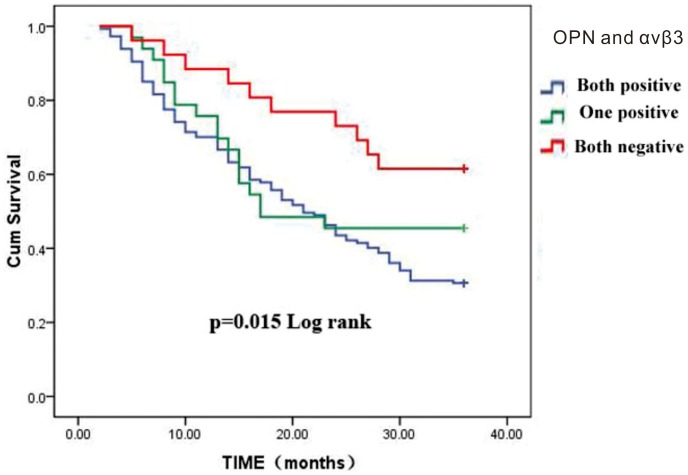
Comparison of the survival time of patients with HCC depending on OPN and αvβ3 expression.

Spearman rank correlation analysis revealed a positive association between OPN and αvβ3 protein expression in HCC specimens (r = 0.29, *P*<0.01) (Table 3). The 1- and 3-year survival rates for patients expressing both OPN and αvβ3 were 69.6% (126/181) and 29.8% (54/181), respectively. In comparison, the 1- and 3-year survival rates for patients negative for OPN and αvβ3 were 86.8% (33/38) and 60.5% (23/38), respectively. The 1- and 3-year survival rates for patients expressing OPN alone were 74.2% (23/31) and 45.2% (14/31), respectively, and 74.6% (41/55) and 47.3% (26/55), respectively, for those expressing αvβ3 alone. Thus, the 1- and 3-year survival rates were lower for patients expressing both OPN and αvβ3 compared with patients negative for both proteins (*P* = 0.015) (Figure 2). Cox regression analysis showed that OPN expression, αvβ3 expression, tumor thrombus of the portal vein, metastasis of the lymph node and clinical staging were independent prognostic factors for poorer survival, while tumor size, capsular invasion were not independent prognostic factors (Table 4).

**Table 3:** Relative protein expression of OPN and αvβ3 in HCC.

OPN	αvβ3		total
	positive	negative	
positive	181	31	212
negative	55	38	93
total	236	69	305

There is a relationship between the expression of OPN and αvβ3 in HCC (P<0.01, x^2^ = 25.42), correlation coefficient, r = 0.29.

**Table 4:** Prognostic analysis of the 305 HCC patients with Cox multivariate regression model.

Variable	B	SE	Wald	Exp (B)	P
OPN	1.679	0.432	15.131	5.362	0.000
αvβ3	1.810	0.439	17.033	6.113	0.000
Tumor size	0.203	0.195	1.083	1.225	0.298
Capsularinvasion	0.108	0.113	0.917	1.114	0.338
Tumorthrombus	1.386	0.478	8.396	3.998	0.004
Lymph metastasis	1.307	0.589	7.934	3.697	0.010
Clinical stage	0.917	0.413	5.437	2.643	0.026

## Discussion

OPN is an extracellular matrix secreted phosphorylated glycoprotein that was originally found by Senger and colleagues [21] in epithelial cells that had undergone malignant transformation. In recent years, additional studies have confirmed that overexpression of OPN can promote tumor growth, invasion and/or metastasis [10]. The expression of OPN in malignant tumors and its relationship with clinical prognosis have been previously reported [22], [23], [24]. In this study, we observed expression of OPN in 69.5% of HCC patients, while expression was significantly lower in adjacent non-tumorous tissue (18.4%, P<0.01), highlighting an important role for OPN in hepatocarcinogenesis. The study has shown that OPN can produce a marked effect by binding to receptors on endothelial cell membranes. Following OPN binding to receptors such as the αvβ3 integrins, it can directly stimulate the differentiation and proliferation of tumour cells, and may regulate the genesis and migration of cancer cells, alter the extracellular matrix, activate intracellular signaling pathways and promote the growth of tumour [15], [25]. The overexpression of OPN allows cells to traverse the G2/M phase turning point, thus conferring malignant and proliferative potency. OPN can also promote the proliferation of endothelial cells, which participate in HCC angiogenesis [26].

In this study, we demonstrate that OPN expression correlates with tumor size, capsular invasion, portal vein tumor thrombus, lymph node metastasis and clinical staging. OPN expression was higher in larger tumors, tumors with capsular invasion, portal vein tumor thrombus and lymph node metastasis. The expression rate of OPN was also significantly higher in patients of clinical stage III, IV compared with stage I and II patients. HCC is highly aggressive, and the prognosis of patients is poor when OPN is highly expressed. In contrast, OPN expression was unrelated to patient gender, age, number of tumors, AFP (alpha-Fetoprotein), HBsAg (Hepatitis B surface antigen), tumor grade and liver function.

High expression of OPN in HCC may be due to its relationship to the cell cycle, angiogenic factors, hormone factors and certain transcription factors. One such candidate factor is αvβ3, an important regulator of gene transcription, closely related to carcinogenesis [10], [11]. In this study, αvβ3 was expressed in 77.4% of HCC, while expression was observed in only 21.6% of adjacent, non-tumorous tissue. This study showed that αvβ3 expression was significantly higher in HCC compared with adjacent normal hepatic tissue (P<0.01), suggesting that αvβ3 may be involved in hepatocarcinogenesis. In addition, αvβ3 protein expression was significantly higher in HCC specimens of clinical stages III and IV with portal vein tumor thrombus and lymph node metastasis compared with phases I and II and tumors without metastasis.

In this study, we demonstrate that expression of OPN is linearly and positively associated with the expression of αvβ3 in HCC. Thus, higher levels of OPN are associated with higher levels of αvβ3. αvβ3 is an important transcription factor, capable of directly promoting angiogenesis and inhibiting apoptosis, by increasing the expression of OPN [27]. It was suggested that OPN acts through αvβ3 integrin, which in turn activates the PI3K/Akt signaling pathways and further contributes to the overexpression of αvβ3 [15].Thus, overexpression of OPN and αvβ3 may have a synergistic effect in promoting hepatocarcinogenesis, jointly participating in proliferation, transformation and regulation of migration in liver cells. We also observed that the 1- and 3-year survival rates of patients positive for both OPN and αvβ3 expression were lower those of patients negative for both proteins. Furthermore, we demonstrated that HCC patients co-expressing OPN and αvβ3 have a poor prognosis by both univariate and multivariate analyses.

In conclusion, expressions of OPN and αvβ3 are significantly higher in hepatocellular carcinoma than in adjacent non-tumorous tissue, and likely play an important role in the development of HCC. The expression of OPN and αvβ3 may also be used as a prognostic indicator of HCC.

## Supporting Information

Figure S1 **Examples of OPN and αvβ3 expression from several patients with high, intermediate, low and negative expression.**
(TIF)
