# Supplementary figures and images for: OPN and αvβ3 Expression are Predictors of Disease Severity and Worse Prognosis in Hepatocellular Carcinoma
Source: PLoS One. 2014 Feb 3;9(2):e87930. doi: 10.1371/journal.pone.0087930 (PMC3912195; doi:10.1371/journal.pone.0087930)

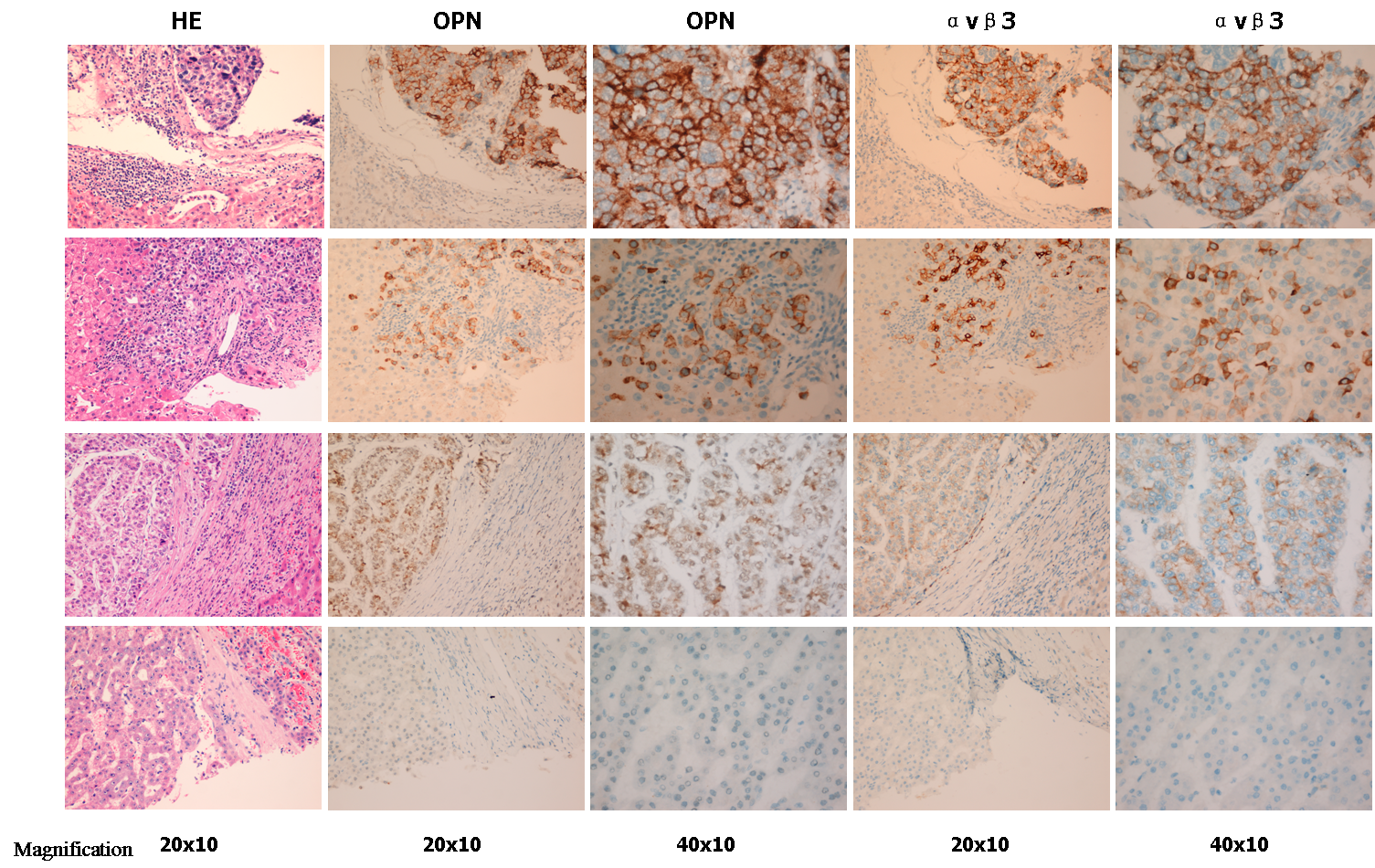

Supplement: Figure S1 — Examples of OPN and αvβ3 expression from several patients with high, intermediate, low and negative expression. (TIF) [file pone.0087930.s001.tif]
